# The Microscope in Relation to Modern Biology

**Published:** 1893-10-07

**Authors:** 


### The Microscope in Relation to Modern Biology.

The present time is essentially the age of machinery, whether in the practical everyday world of industry and manufacture, or in the secret recesses of the laboratory of the student. And just as innumerable articles, which can now be easily and rapidly turned out, would, a few years ago, have been impossible, or could only have been made with extreme difficulty, so also many scientific researches are now placed within the reach of any one who possesses ordinary manipulative skill, which ten years ago would have taxed to the utmost all the abilities of the most accomplished investigator.

And of these appliances, which are now at the disposal of every student of science, few have been improved so much as the microscope, and the accessory apparatus which is necessary for the adequate use of this most important instrument. It is now more than two hundred years since Robert Hooke, an Englishman, first clearly saw and gave the name of cells to those minute particles of which animals and plants are built up. He was speedily followed by a number of workers in the same field, and it is really astonishing to turn back to those old books, and witness the fidelity with which such men as Malpighi and Nehemiah Grew pourtrayed, with the aid of the rude and imperfect microscopes at their command, the minute structure of the tissues which they investigated.

The workers of this old time are most instructive also from another point of view, since they show clearly that although the workman must have his tools in order to labour at all, the use to which he puts them for the most part rests with himself. After the splendid beginnings made by the school represented by the men above named, a long period of sterility intervened, in which hardly anything worth retaining was produced. Not, indeed, that observations and the publication of bulky volumes were lacking, but the practical value of the energy thus spent amounted to almost nothing, except in so far as it served to indicate the kind of attitude which it is necessary to avoid, in pursuing all sound scientific inquiry. The period to which we refer is that in which the scholastic philosophy claimed as its votaries those who should have been devoting themselves to laying the foundations of truth, upon which a future generation might continue to build. Instead of this, the method they followed led them to substitute fiction for fact, ideas for realities. One has only to turn to the works of the speculative biologists of the early part of the last century to see the effect of this bias of mind; but, fortunately, like most other evils, it ended by stirring up an outcry against itself, and thus the course of research became once more directed into fruitful channels, and the scientific method was permanently introduced into histology, as it had been already into other branches of science. Many people talk of "Scientific Method" as if it were something foreign to the ordinary processes of thought, but it really means nothing more than putting the cart behind, instead of before, the horse—drawing one's conclusions from facts, instead of facts from conclusions.

When the minute structure—histology as this branch of anatomy is generally called—became once more the object of earnest research, it was not long before striking similarities between animals and plants were detected. The gelatinous substance—sarcode—of which the soft parts of animals are composed, was seen to have its counterpart in the slime which filled the interior of the vegetable cell. Further than this, about sixty years ago, and chiefly through the researches of Schleiden and Schwann, the regular presence of a structure called the nucleus, in the protoplasm of each cell in the bodies of both animals and plants, became securely established. It is true that these men were not the first to recognise this structure, nor did they rightly apprehend its real nature, but still to them belongs the credit of having first insisted upon its paramount importance.

Finally, about the middle of the present century Von Mohl gave the name "Protoplasm" to the vegetable slime, and some years later Cohn and Max Schultze identified the animal sarcode with it, and Protoplasm, the physical basis of life, as Professor Huxley has called it, is now the name by which the living substance of animals and plants is universally known. It is hardly surprising that histological research during the last few years has been mainly directed to the elucidation of the structure of the *cell*, which consists essentially of the protoplasm and its contained nucleus. The framework, or cell-wall, in an ordinary vegetable cell, for instance, is not really of vital importance; it may or may not be present, and, indeed, is commonly wanting in animals.

The nucleus has received a large share of attention, for most biologists now believe that those forces which direct the course of development of the cells, and which we collectively group under the term heredity, are centred in it. And we now know that during the formation of fresh cells, which always arise from pre-existing ones, it is in the nucleus that the most striking changes occur—changes, indeed, so extraordinary, and of so complicated a nature, that many volumes have been written describing and attempting to explain them.

But these delicate researches can only be conducted by utilising all the resources which lie at the disposal of the investigator. As the effects of the re-agents and stains which are employed in work of this kind, become better known, the observations can be more skilfully conducted, more carefully checked, and more consistently explained. Many persons seem to think that it is an easy task to look down a microscope and to describe what one there sees, but a very slight practical acquaintance with the subject soon suffices to dispel such an idea. One ceases then to wonder at the disagreement between well-known writers on actual matters of fact, as well as at the apparent facility with which views once firmly held come to be abandoned. In fact, there is hardly any study which calls for such constant caution, alertness, and judgment, as microscopic research.

Valuable assistance is now being afforded by the introduction of photography in connection with studies of this nature, especially where high magnifying powers are concerned. By its aid the microscopic image is, so to speak, published, and the conclusions drawn from it are thenceforward open to general criticism. But notwithstanding that its importance has already been fully recognised in some branches of biology, such for example, as bacteriology, it has not as yet experienced that general appreciation which it will certainly one day meet with. One reason, quite apart from the actual difficulties of securing the photograph, lies in a misapprehension which exists, even amongst actual workers, as to the functions of a photo-micrograph. They prefer a drawing, executed by the observer, as combining in one clear picture the impressions arrived at by working through a number of preparations; the microscope shows so much, that it is difficult to disentangle the essential from the accidental or superfluous in any one slide. But the obvious answer is that just as a drawing should be an interpretation, the photograph should be regarded as the representation, of the facts on which the interpretation rests. They each have their proper functions, and these functions are distinct. J. B. F.

